# Control of Body Weight by Eating Behavior in Children

**DOI:** 10.3389/fped.2015.00089

**Published:** 2015-10-19

**Authors:** Modjtaba Zandian, Cecilia Bergh, Ioannis Ioakimidis, Maryam Esfandiari, Julian Shield, Stafford Lightman, Michael Leon, Per Södersten

**Affiliations:** ^1^Section of Applied Neuroendocrinology, Karolinska Institutet, Mandometer Clinic Novum, Huddinge, Sweden; ^2^Department of Paediatric Endocrinology, School of Clinical Sciences, University of Bristol, Bristol, UK; ^3^Department of Diabetes, School of Clinical Sciences, University of Bristol, Bristol, UK; ^4^Department of Metabolic Endocrinology, School of Clinical Sciences, University of Bristol, Bristol, UK; ^5^Henry Wellcome Laboratories for Integrative Neuroscience and Endocrinology, Department of Medicine, University of Bristol, Bristol, UK

**Keywords:** children, body weight, brain, genes, eating behavior

## Abstract

Diet, exercise, and pharmacological interventions have limited effects in counteracting the worldwide increase in pediatric body weight. Moreover, the promise that individualized drug design will work to induce weight loss appears to be exaggerated. We suggest that the reason for this limited success is that the cause of obesity has been misunderstood. Body weight is mainly under external control; our brain permits us to eat under most circumstances, and unless the financial or physical cost of food is high, eating and body weight increase by default. When energy-rich, inexpensive foods are continually available, people need external support to maintain a healthy body weight. Weight loss can thereby be achieved by continuous feedback on how much and how fast to eat on a computer screen.

## Introduction

The medical consequences of overweight and obesity in adults and children alike are well known (1–3), and although attempts to curb further escalation of the problem have met with some limited success (4–7), childhood obesity continues to increase throughout the world (8). Given that the biological mechanisms that contribute to weight regulation are complex (9–12) it is a challenge to treat obesity (13–15).

Public health initiatives, e.g., making people eat low-calorie foods and banning fast food commercials (16–18) and/or increasing their exercise level, e.g., implementing exercise programs at school (6, 16, 19), have also met with some success. Most attempts at developing obesity drugs have been abandoned owing to unwanted adverse effects (20); drug-induced body weight loss is most often <6% (21–23). New hope for weight-loss drug development has been ignited by “precision medicine” (24). This approach, which is based on an understanding of individual genetics, has been successful in managing some forms of cancer; the question is whether it can be used to develop individualized drugs to treat obesity, given the complexity of the interaction between genes and environment in human behaviors (25–27).

What is known, however, is that gastric surgery reduces body weight of both adults and children (28). Interestingly, the effect in obese mice appears not to be due to the decrease in gastric size, but to an increase in bile acids and their associated changes in intestinal microbe populations (29). Mimicking these effects pharmacologically might work with obese humans, and may even prevent the re-emergence of type-2 diabetes, which has been reported after bariatric surgery, despite the maintenance of a reduced body weight (30, 31). Although mimicking the physiology of weight loss with drugs has proved to be an ineffective strategy, it may be possible to mimic the change in bile acids to induce weight loss, a possibility supported by an impressive, comprehensive analysis of this subject (32).

We suggest, however, that the reason why most approaches to effect weight loss have failed is that the causes of obesity have been misunderstood and that our present understanding of how the neuroendocrine system is engaged in eating and body weight regulation is mistaken.

## You are *Not* What You Eat

Although it was reported that counseling aiming at reducing caloric intake and increasing physical activity in two large groups of overweight and obese patients had negligible effects (33, 34), it was observed that behavioral factors, rather than macronutrient composition, are the main influences on weight loss. Some support for modifying eating behavior to induce weight loss was noted in a large-scale project with children (35, 36), but compelling results have not yet been published from this project. A recently published meta-analysis of the available data indicated that diet and exercise interventions have very small, albeit possibly clinically significant, effects on body weight (37). The specific content of diet appears unimportant, but what is important is patient compliance (36). However, it is axiomatic that those who are able to eat less food will lose weight and that those who are not able to do that, will not lose weight. Although some have found that an increase in physical activity combined with a reduction in calorie intake can result in a modest decrease in body weight (37), this feat is rarely accomplished. Moreover, others have concluded that calorie restriction with increased exercise is no better than calorie restriction alone (38).

## The Brain Does Not Control Eating to Maintain a Constant Body Weight

More than 100 years ago, it was discovered that hypothalamic damage is associated with changes in body weight. The subsequent experimental research on this topic concluded that there are dual hypothalamic centers that either induce or inhibit eating to maintain a constant body weight (39). A similar model of body weight control remains today, with the exception that while excitation and inhibition of eating behavior were originally thought to be only anatomically separable, they are now considered to be both chemically and anatomically distinct (40, 41). Body weight is thought to be kept stable through the activity of orexigenic and anorexigenic signaling molecules that respond to feedback from adipose tissue, the pancreas, and the gastrointestinal tract (42). But rather than being maintained at a constant level homeostatically, body weight is drifting upward all over the world – hypothalamic anorexigens that signal satiety are clearly unable to arrest this development.

The problems with the hypothalamic model of food intake emerge when attempts are made to translate this concept into the clinic. Thus, most purported orexigens, which should increase food intake, are elevated in patients with anorexia nervosa, yet these patients rarely eat (43). And when anorexic patients eat more food, their blood levels of an anorexigen, such as leptin, increases (44). An inhibitor of food intake, of course, should not be found at high levels at a time of increased food intake. Conversely, since obese patients have elevated levels of the purported anorexigen leptin, and they continue to eat large amounts of food (45), leptin cannot be an anorexigen. To rescue the concept of orexigens/anorexigens, it has been suggested that obese patients are insensitive to leptin. However, insensitivity to leptin is a fragile idea because: (1) the sensitivity to leptin can be restored with amylin treatment, (2) the brains of experimental animals eating a high-fat diet are as sensitive to leptin as are the brains of animals eating a low-fat diet, and (3) while sensitivity to leptin can be suppressed in one brain area, it can be retained in another (46).

To some extent, therefore, these conceptual problems are created by the outdated hypothalamic homeostatic framework. It would be very surprising indeed if the cause of obesity is found in the presumably abnormal brains of the hundreds of millions of people who are now obese. Their brains are probably simply doing what they have evolved to do, and the cause of their problem is more likely outside than inside of their body (47). Body weight was kept low over millennia through the lack of easily available food (48). Hence, all animals, including humans, have evolved efficient behavioral strategies foraging for food (49), and these strategies can be studied experimentally. Thus, by dissociating the behavioral responses for foraging for food (appetitive behavior) from those employed for eating food (consummatory behavior) in laboratory animals, it was discovered that neuropeptide tyrosine, a “hypothalamic orexigen,” stimulated the search for food at the expense of eating, and that leptin, generally recognized as an inhibitor of eating, markedly increased eating when the need to search for food was circumvented (50). These results are inconsistent with the hypothalamic homeostatic framework, but consistent with the behavioral and clinical phenotypes of underweight and overweight patients (51).

However, a remarkable recent demonstration that activation of a mere 800 hypothalamic neurons evoked voracious feeding within minutes in mice compelled the authors to conclude that these neurons serve a “dedicated role coordinating this complex behavior” (52), inducing renewed optimism: “the future of hypothalamic research is clearly bright” (53). However, this conclusion was rapidly challenged by the even more recent finding that the same cell groups were activated immediately upon presentation of food to a mouse, but before it started to eat, persuading these authors to suggest that these neurons were involved in appetitive, rather than consummatory behaviors (9, 42), confirming the findings of Ammar et al. (50). However, the authors were perhaps inaccurate in suggesting that the neurons that were studied actually control appetitive behavior. Instead, it was clear that the neurons responded to the presentation of food, and as a consequence, the animal engaged in appetitive behavior; the neurons were controlled by the environment. The importance of such neurobiological adaptation to environmental changes as related to eating has long been known (54, 55).

These recent studies support the hypothesis that the brain permits, rather than controls eating, and that eating and body weight are controlled externally, with the major regulatory factor being the physical and economic price of the food (47, 48, 51, 56, 57). Because hypothesized hypothalamic orexigens can turn into anorexigens in response to a change in the environment (48), drugs developed to decrease eating might actually increase eating when that change occurs. For this reason, the utility of the pharmacological approach to the control of eating behavior and body weight will probably continue to be minimal (58, 59).

## Enter Precision Medicine

It has recently been suggested that obesity is the result of a chronically disordered brain (60). On this perspective, the brain is thought to reprioritize behavior outputs and increase food intake independent of energy stores in the form of body fat (61). More plausibly, eating a great deal of food should be considered to be a normal response to palatable, easily accessed food (47, 62). Encouraging people to make healthy food choices (63) should therefore be expected to fail.

The risk of obesity is also thought to be due in part to genetic factors (61). According to a recent report, 97 gene loci are related to the control of body weight, including genes involved with specific pathways in the brain that engage multiple neurotransmitter systems (64). In order to take all of these factors into consideration, precision medicine would have to develop perhaps hundreds of drugs to reliable produce weight loss. The promise that this approach will solve most problems related to obesity seems unlikely.

However, we know that the major factor that prevented an increase in body weight throughout the world was the poor availability of food (49). Humans also evolved to run for long distances to be able to capture wild game to support their life, but today such routine exercise is rarely part of daily life, impacting negatively on health (65). It has been suggested that the search for the candidate genes for the human homeostatic phenotype that would allow the maintenance of a constant body weight should consider the ways in which glycogen and triglycerides are stored in skeletal muscle, a key to optimal physical activity (66). Skeletal muscle fitness has since been elegantly analyzed and has been associated with both cardiovascular and mental health (67, 68). However, although exercise genomics as it relates to metabolic health is making impressive progress (69), even the genes of the world’s best runners (70) do not protect their carriers from the effects of living in an easy food environment (71).

## You are *How* You Eat

Thus, our brain and genes permit rather than control eating, and because evolution has not encouraged satiety, we eat in most circumstances, and unless we have to work for the food, our body weight increases (51). Unsurprisingly, attempts at dieting also increase, but rather than having the desired effect, dieting increases the risk for losing control and gaining even more weight (72). We have suggested, therefore, that today when inexpensive high-energy foods are continually available at low cost, we need external support to eat a normal amount of food and maintain a healthy low body weight (47).

A treatment for childhood and adolescent obesity that relies on this perspective was an adjustment of a treatment for underweight patients, who had been diagnosed with anorexia nervosa. Addressing their disordered eating behavior directly was shown to be effective in a randomized controlled trial with a remission rate of 75% in on average 1 year of treatment and a rate of relapse of 10% over 5 years of follow-up (73, 74). Anorexia typically develops at 14–19 years of age and the patients in our studies were on average 17.5 years old, having been diagnosed on average 3.2 years earlier (74). However, it is important that the treatment is effective in most age groups, and therefore, age is not an exclusion criterion.

A critical part of therapy is giving them mealtime feedback on their speed of eating. Anorexics eat very little food very slowly, and they use the feedback to practice eating more food at a higher speed. The feedback shows their speed of eating on a small monitor next to the normal rate of consumption of such a meal. In an effort to treat obese adolescents and children, this same approach was used, with the exception that the rapid food intake of such individuals was normalized with the mealtime feedback (75). The underlying assumption is that anorexia and obesity are the extreme opposites with the same behavioral problem; both have lost control over the speed at which they eat their food (51). It should be noted, however, that although eating quickly is associated with overconsumption of food (76), it has not yet been demonstrated quantitatively that the obese actually eat quickly. And if they do, the cause–effect relationship between eating speed and obesity remains to be determined.

**Figure 1:**
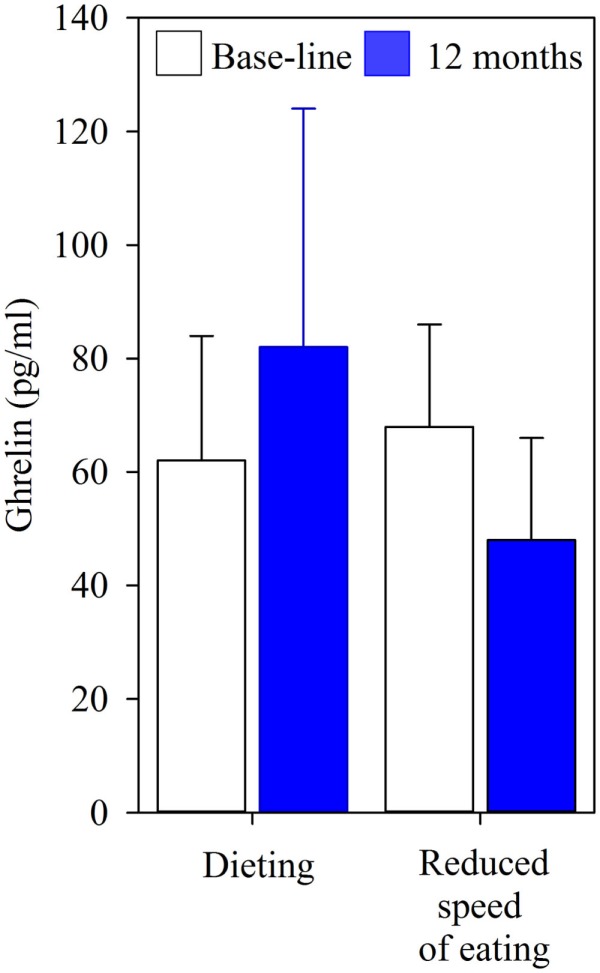
**Fasting concentrations of ghrelin in 13 adolescents who received a standard diet intervention for 12 months, and in 14 who practiced eating at a reduced speed for 12 months**. The children were on average 12.5 years old, the values are mean (SD).

A randomized controlled trial showed that reducing the speed of eating decreased the food intake and the body weight of obese 12.5 years old more effectively than a standard diet intervention, and, interestingly, the effect was maintained over 6 months of follow-up (75). Relapse into obesity, which is all too common, is hypothetically caused by irreversible biological changes, such as elevated levels of ghrelin, a purported hunger hormone (11, 77). However, treating eating behavior directly reduced, rather than increased the level of ghrelin [Figure 1 (78, 79)]. Changes in endocrine secretions through experimentally induced changes in behavior, such as the effect of an alteration in eating behavior on ghrelin, show that this principle can be translated into the clinical management of childhood obesity.

**Figure 2:**
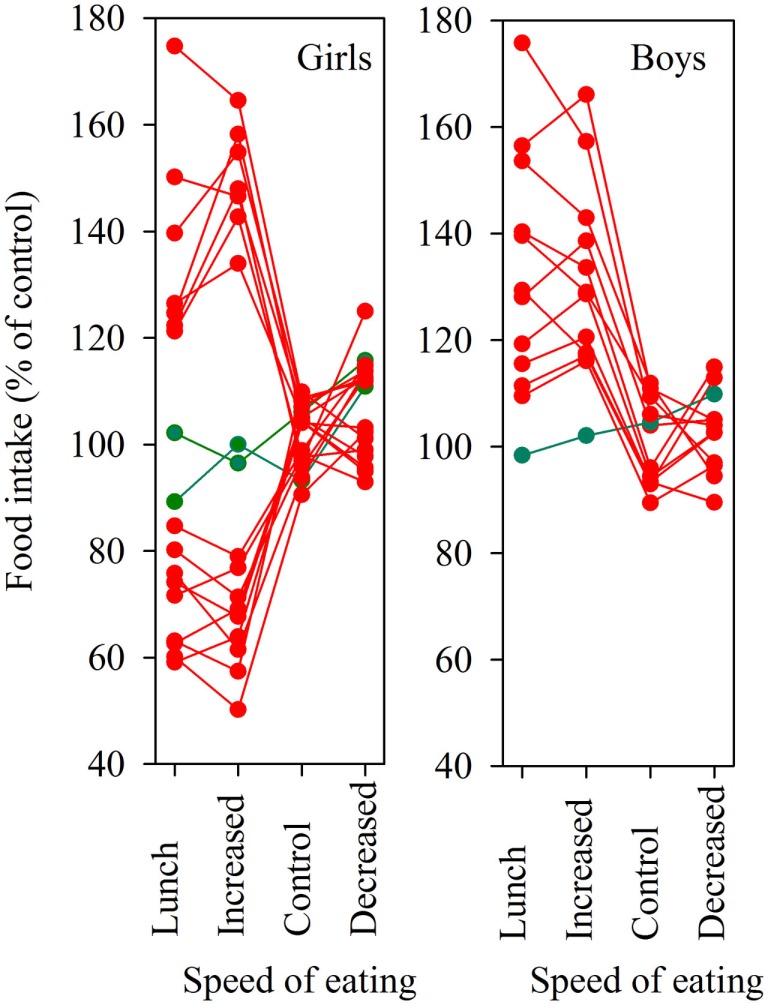
**Food intake during the school lunch in 18 girls and 12 boys**. The children had their speed of eating experimentally increased, unchanged, or decreased in relation to its value in a meal eaten without constraints, the values are expressed as percent of that value. Green lines indicate individuals able to maintain their food intake independent of the speed of eating. The children were on average 13 years old.

A change in eating behavior can be caused by an environmental change. For example, the limited amount of time available for eating lunch forces school children to eat quickly. Thus, 100, on average 13 years old, children spent on average just 7 min eating their school lunch with their peers (80). A more detailed study showed that 18 girls ate their lunch in as little as 5.6 min and 12 boys ate theirs in a mere 6.8 min. Only two girls and one boy were able to maintain their food intake at the level observed when they ate individually (258 g in girls and 289 g in boys). Nine girls ate on average 33% less food and seven girls ate 23% more food, whereas the remaining boys ate 26% more food when eating their school lunch rapidly with their class mates (Figure 2). The average speed of eating increased to 183% in the girls and to 166% in the boys compared to when they were eating individually. These changes in food intake during school lunches were replicated by experimentally increasing the speed of eating when the children were eating individually (Figure 2) (80). Thus, most boys and half of the girls eat too much when forced to eat quickly; only a few are able to eat a normal amount of food.

The main risk factor for loss of control – dieting – also causes a sexually dimorphic change in eating behavior. Although men who skipped dinner eat more food quickly the next day, women eat less food the next day at a constant speed (81). If maintained, this pattern of eating results in a further loss of control; when women who eat at the same speed throughout their meal are forced to eat quickly, they over-eat. Conversely, when forced to eat slowly, they under-eat (82). They rapidly adopt the pattern of eating characteristic of obese patients and anorexics, who both eat at a constant speed (83). Interestingly, using the feedback method discussed above, anorexic and obese patients are able to eat normal-size meals at the normal speed, suggesting that the biological changes associated with these individuals do not prevent them from eating normally (81, 82).

## Concluding Remarks

It is probably normal to eat large quantities of food when eating is easy. Most people, therefore, need external support to eat a proper amount of food and maintain a healthy low body weight. Such support can be provided by feedback on a computer screen to both under- and over-weight patients. This method has brought hundreds of patients with eating disorders into remission (74) and is presently used effectively with obese children and adults. It must be added, however, the effects of new methods need to be further evaluated and followed up before they are implemented into health-care systems.

## Author Contributions

MZ, II, ME, JS, and SL performed experimental work. PS and CB developed treatment and technology. PS and ML wrote the paper. PS authorized by authors for the final version of the manuscript.

## Conflict of Interest Statement

Per Södersten and Cecilia Bergh own 47.5% each of the stock in Mando Group AB, Michael Leon owns 5%. Mando Group AB holds the IPR of Mandometer, the FDA-approved medical device used to treat patients with eating disorders in clinics managed by Mando Group AB. Swedish health care is publically funded. Modjtaba Zandian, Ioannis Ioakimidis, and Per Södersten are appointed by Karolinska Institutet, all salaries are paid by Mando Group AB. Ioannis Ioakimidis is supported by EU-grants.

## Funding

This work is supported by Mando Group AB.
